# Consider systemic capillary leak syndrome in monoclonal gammopathy with shock

**DOI:** 10.1016/j.amsu.2021.103013

**Published:** 2021-11-02

**Authors:** Ounci-Essad, Amine Bouchlarhem, Oussama Lamzouri, Ghizlane El aidouni, Leila Haddar, Hamza Mimouni, Houssam Bkiyar, Brahim Housni

**Affiliations:** a Faculty of Medicine and Pharmacy, Mohammed I^st^ University, Oujda, Morocco; b Department of Anesthesiology and Intensive Care Unit, Mohammed VI University Hospital, Mohammed I University, Oujda, Morocco; c Mohammed First University, Faculty of Medecine and Pharmacy, LAMCESM, Oujda, Morocco

**Keywords:** Cappilary leacking syndrome, Hypovolemic choc, Hypoalbuminemia, Hemoconcentration, Monoclonal gammapathy, Clarckson syndrom

## Abstract

**Introduction and importance:**

The cappilary leacking syndrome is a very rare disease that can be idiopathic (clarkson syndrome) or secondary to other pathologys.

**Case presentation:**

We report a case of 37-year-old women who was admitted in the emergency room for a hemodynamic shock of neither cardiac nor septic cause, and the patient wasn't presenting any bleeding. The investigations showed that the diagnosis was a Clarkson syndrome crisis and the patient was having supportive treatment containing fluid therapy, vasoactive drugs, and ECMO. And died after 48h of hospitalization.

**Clinical discussion:**

the cappilary leacking syndrome is a very fatal affection, its physiopathologis remains unknown. It evoluate by crisis made by hypotension and anasarca, in severe cases it is presented as fatal hypovolemic schock. Biological investigations show hemoconcentration associated with hypoalbunemia which is pathognomonic of the disease. The treatment is essentially based on crisis treatment support by fluid therapy, vasoactives drugs, some practicien report the use of theophilyn for prevention but without any proven efficiency.

**Conclusion:**

For all this reasons we are in the obligation of investing in fundamental studies to better understand this fatal disease.

## Highlights

• The cappilary leacking syndrome is a very rare disease.
• The physiopathology of cappilary leck syndrom remains unknown.
• It evoluate by crisis made by hypotension and anasarca, in severe cases it is presented as fatal hypovolemic schock.
• Hemoconcentration associated with hypoalbunemia is pathognomonic of the disease.

## 1 Introduction

The cappilary leacking syndrome is a very rare cause of hemodynamic shock that is life threatening, few cases were reported, and actually this disease remains a real problem for practicians for its high mortality rate. The clinical presentation is marked by the appearance of recurrent episodes of effusions that can lead to fatal hemodynamic shock. We report a case of 37 years old patient having a history of five episodes of hypotension associated with anasarca admitted for hemodynamic schock state ibecause of cappilary leacking syndrome.

## 2 Case presentation

We report the case of a 37-year-old woman, with a history of 5 episodes of hypotension with generalized edema spontaneously reversible in 2 years, admitted for the management of asthenia with edema of the lower limbs reaching the thigh for 4 days with a recent weight gain estimated at 8kg in 2 days. The initial evaluation found patient, hemodynamically unstable with unfound blood pressure and Heart Rate = 157 bpm, cold extremities, an elongated refilling capillary time, respiratory, FR = 34 cpm with SpO2 = 97% on room air, G = 1.17 and T° = 36.7, the patient is oligo-anuric.

The physical examination found a patient in a state of complete anasarca with no signs of right heart failure, cardiac examination is without particularities apart from tachycardia, pleuropulmonary auscultation finds bilateral snoring rales.

Vascular filling with 3 L of 0.9% saline solution is performed without hemodynamic or renal response requiring the introduction of Norepinephrine with a dose of 0.4 μg/kg/min after taking a central venous approach with an initial central venous pressure (CVP) estimated at 1 mmhg.

An electrocardiogram (EKG) was performed and did not show any repolarization disorders, on transthoracic echocardiography there were no signs of acute pulmonary heart, nor any segmental or global kinetic disorders that could explain this picture, the left ventricualr ejection fraction (LVEF) was estimated at 50%, the inferior vena cava was collapsed. The biological assessment performed on admission showed: Hemoglobin at 19.7 g/dl, Hematocrit at 69%, creatinine at 21mg/l, urea at 0.97 and albumin at 16 g/l with a normal infectious work-up and negative proteinuria (Table 1). The arterial blood gaz on admission showed a non-compensated metabolic acidosis with hyperlactatemia at 7.37 mmol/l (Table 2).

**Table 1:** Biological findings.

Variables	Reference range	H0	H10	H24	H36	H40
Complete blood cell count						
White blood cells (/ul)	4000–10000	6580	7500	6300	9600	104,000
Segment neutrophil (/ul)	1500–7000	4670	5320	5019	4870	5309
Lymphocyte (/ul)	1000–4000	1760	1540	2013	1875	1748
Hemoglobin (g/dl)	12–16	19,7	20.3	21.4	19.4	20.1
Platelets ( × 10^4^/μl)	15–40	21.9	14,7	10,34	5,34	4,29
Hematocrit (%)	37–49	69	72,3	70	67.9	78,3
Blood chemistry						
Sodium (mmol/l)	135–145	147	145	147	142	138
Potassium (mmol/l)	3.5–5.0	3.7	4,9	5,0	5,3	5.3
Glucose (g/l)	0,7–1,05	1,17	1,22	1,03	1,21	1,13
C-reactive protein (mg/l)	0–5	17,87	26.24	20.77	____	___
Procalcitonin (ng/ml)	0.00–0.05	0.01	0.015	___	___	___
Total protein (g/d)	64–83	34,7	32,8	31,8	29,8	30,6
Albumin (g/l)	35–50	16	15,87	15,2	15,23	14,76
Blood Urea nitrogen (g/l)	0,15–0,45	0,97	1,51	2.24	1,8	2.15
Creatinine (mg/l)	6–11	21	49,98	73,65	51.71	59.62
Total bilirubin (mg/l)	4–16	4,76	7,54	5,98	14,89	16,23
Aspartate aminotransferase (U/l)	5–34	57	65	78	138	142
Alanine aminotransferase (U/l)	0–55	65	69	86	102	172
Alkaline phosphatase (U/l)	40–150	149	175	168	242	265
Lactate dehydrogenase (U/l)	124–222	598	571	410	452	438
Creatinine kinase (U/l)	60–250	201	652	765	1203	1394
Blood coagulation						
Prothrombin time (%)	75–100	76	84	64	52	35
partial thrombin time (sec)	24–29	24	27	32	42	53
Fibrinogen (g/l)	2–4	2,6	5,4	6,3	5,8	6,1
D-dimer (mg/l)	＜0,5	0,35	0,49	0,75	1,5	2,8

**Table 2:** Blood arterial gaz result.

Variables	Reference range	H0	H9	H24	H36	H48
pH	7,37–7,43	7,04	7,09	7,14	6,98	6,87
Pao2 (mmhg)	60	78,7	146	172	174	169
PAco2 (mmhg)	35–45	20,7	79,8	40,7	39,9	30,8
HCO3- (mmol/l)	22–26	5,98	7.3	10,8	6,98	3,8
Hb (g/dl)	13–17	21.9	19,7	20,3	18,6	19,76
SaO2 (%)	94	91	93	90	94	89
Lactate (mmol/l)	0–2	7,64	12,8	9,72	14,78	15,98

Given the absence of criteria for cardiogenic or septic shock and the absence of active bleeding, the history of 5 recurrent episodes of hypotension with generalized edema and the presence of hemoconcentration and low albumin in this young patient, the diagnosis of clarckson's syndrome was evoked.

Massive filling with macromolecules (hydroxyethylamidon) is initiated 9 hours after admission, the patient began to drop down blood pressure, requiring augmentation of norepinephrine with the introduction of dobutamine with no hemodynamic response.

The patient presented a cardiac arrest that have been recovered with a NO FLOW at 0 and a LOW FLOW at 13min, the patient was intubated and then hemodynamically stabilized with epinephrine with a Blood Pressur: 90/50 mmhg. The evolution is marked by the clinic-biological aggravation of the renal function requiring the introduction of a continuous replacement therapy of the Kidney, with the installation of a rhabdomyolysis.

The electrophoresis of the blood proteins found a monoclonal peak of type IgG kappa in favour of our diagnostic hypothesis, the microbiological cultures were negative. Blood gas monitoring always showed an uncompensated metabolic acidosis with hyperlactatemia.

48h after her admission, the patient started to drop her blood pressure BP = 60/30 mmhg, given the refractory shock state, the implementation of circulatory assistance by a veno-arterial Extra Corporeal Membran Oxygenation (ECMO) is decided but unfortunately the patient presented a non-recovered cardio-respiratory arrest a few moments later.

## 3 Discussion

The capillary leakage syndrome first described in 1960 [1]. Can be idiopathic (clarkson syndrome) or secondary to several pathologies [2].

It is a very rare syndrome. Since the first description, more than 150 cases have been reported in the literature, mostly as isolated cases with limited follow-up.

The clinical presentation is usually weight gain with generalized edema sparing the lung and may lead to ENT (ear-nose-throat) or digestive signs in connection with mucosal edema with oligoanuria and hypotension that may be complicated by hemodynamic shock.

Biologic investigation shows, an hemoconcentration with paradoxical hypoalbuminuria is pathognomonic with functional renal failure. Protein electrophoresis (PPE) very frequently shows monoclonal immunoglobulin (mainly IgG) (away from crisis, except for PPE, which remains abnormal, the other tests are normal).

The pathophysiology is not well understood, but pathophysiological hypotheses have been put forward, notably the role of inflammation in the initiation of attacks, although the role of cytokines has not been demonstrated [3].

The role of monoclonal gammopathy has been discussed but their level remains unchanged between attacks. An increase in endothelial permeability has been demonstrated in an in vitro model of endothelial cell culture after addition of serum collected during attacks. However, capillary permeability was not altered by immunoglobulin purified from patient serum, as well as was the serum collected outside of seizures in this experimental model.

Cadherin internalization and actin fiber formation are evidence of a vascular stress state altering capillary permeability, vascular endotheal growth factor (VEGF) and angioprotein 2 were higher during seizures justifying that treatment by an angioprotein 2 inhibitor and intravenous immunoglogbulin significantly decreasing capillary permeability while the VEGF inhibitor (bevacizumab) exerted a minimal effect [4].

Since the first description of Clarkson's syndrome several treatments have been tried, initially beta mimetic treatment combined with theophylline was administered without consistent results, Recently, intra venous Immuno Globulin (IVIG) treatment for seizure prophylaxis at a dose of 2g/kg/month over 48 hours has reduced the frequency and severity of seizures and is recommended by the authors of this study of patients in the European registry [5,6]. In addition, IVIG treatment may be effective during attacks according to limited data [7].

This case is written following the SCARE guidelines [8].

## 4 Conclusion

We hope that fundamental researches can study more cases of this pathology and make advances that can deliver the diagnostic, prognostic, and therapeutic tools sorely needed to combat this devastating disease.

## Provenance and peer review

Not commissioned, externally peer-reviewed

## Sources of funding

None.

## Ethical approval

The ethical committee approval was not required give the article type (case report). However, the written consent to publish the clinical data of the patients was given and is available to check by the handling editor if needed.

## Consent

Written informed consent was obtained from the patient for publication of this case report and accompanying images. A copy of the written consent is available for review by the Editor-in-Chief of this journal on request.

## Author contribution

Ounci Es-saad: study concept or design, data collection, data analysis or interpretation, writing the paper. Amine Bouchlarhem: data analysis or interpretation, writing the paper. Oussama Lamzouri: data analysis or interpretation, writing the paper. Leila Haddar: data collection. Hamza mimouni: data collection. Houssam bkiyar: supervision and data validation. Brahim Housni: supervision and data validation.

## Registration of research studies

This is not an original research project involving human participants in an interventional or an observational study but a case report. This registration is was not required.

## Guarantor

Ounci Es-Saad.

Amine Bouchlarhem.

## Declaration of competing interest

None.

## References

[1] Clarkson B., Thompson D., Horwith M., Luckey E.H. (1960). Cyclical edema and shock due to increased capillary permeability. Am. J. Med..

[2] Duron L., Delestre F., Amoura Z., Arnaud L. (2015). Syndrome de fuite capillaire idiopathique et formes secondaires : une revue systématique de la littérature [Idiopathic and secondary capillary leak syndromes: A systematic review of the literature]. Rev. Med. Interne..

[3] Cicardi M., Gardinali M., Bisiani G., Rosti A., Allavena P., Agostoni A. (1990). The systemic capillary leak syndrome: appearance of interleukin-2-receptor-positive cells during attacks. Ann. Intern. Med..

[4] Xie Z., Ghosh C.C., Patel R., Iwaki S., Gaskins D., Nelson C. (2012). Vascular endothelial hyperpermeability induces the clinical symptoms of Clarkson disease (the systemic capillary leak syndrome). Blood.

[5] Amoura Z., Papo T., Ninet J., Hatron P.Y., Guillaumie J., Piette A.M. (1997). Systemic capillary leak syndrome: report on 13 patients with special focus on course and treatment. Am. J. Med..

[6] Kapoor P., Greipp P.T., Schaefer E.W., Mandrekar S.J., Kamal A.H., GonzalezPaz N.C. (2010). Idiopathic systemic capillary leak syndrome (Clarkson's disease): the Mayo clinic experience. Mayo Clin. Proc..

[7] Lambert M., Launay D., Hachulla E., Morell-Dubois S., Soland V., Queyrel V. (2008). High-dose intravenous immunoglobulins dramatically reverse systemic capillary leak syndrome. Crit. Care Med..

[8] Agha R.A., Franchi T., Sohrabi C., Mathew G., for the SCARE Group (2020). The SCARE 2020 guideline: updating consensus surgical CAse REport (SCARE) guidelines. Int. J. Surg..

